# Trajectories of functional impairment in homeless older adults: Results from the HOPE HOME study

**DOI:** 10.1371/journal.pone.0221020

**Published:** 2019-08-13

**Authors:** Rebecca T. Brown, David Guzman, Lauren M. Kaplan, Claudia Ponath, Christopher T. Lee, Margot B. Kushel

**Affiliations:** 1 Division of Geriatrics, University of California San Francisco, San Francisco, California, United States of America; 2 Division of Geriatric Medicine, Perelman School of Medicine at the University of Pennsylvania, Philadelphia, Pennsylvania, United States of America; 3 Corporal Michael J. Crescenz Veterans Affairs Medical Center, Philadelphia, Pennsylvania, United States of America; 4 Division of General Internal Medicine, University of California San Francisco, San Francisco, California, United States of America; 5 Zuckerberg San Francisco General Hospital and Trauma Center, San Francisco, California, United States of America; 6 UCSF Center for Vulnerable Populations, San Francisco, California, United States of America; 7 Resolve to Save Lives, New York, New York, United States of America; University of Malaya, MALAYSIA

## Abstract

Difficulty performing activities of daily living (“functional impairment”) is common in homeless adults aged 50 and older. However, little is known about the trajectory of these impairments, nor the extent to which these trajectories are similar to those of older adults in the general population. We identified trajectories of functional impairment in homeless adults aged 50 and older, and risk factors for differing trajectories. We conducted a prospective cohort study of 350 homeless adults, aged 50 and older, recruited via population-based sampling in Oakland, California and interviewed at 6-month intervals for up to 3 years. We assessed functional trajectories based on self-reported difficulty performing 5 activities of daily living. We used multivariable multinomial logistic regression to identify baseline risk factors for each trajectory. At baseline, participants’ mean age was 58 years (SD, 5.3), 24.1% were women, 80.9% were African American, and 38.6% had difficulty performing 1 or more activities of daily living. We identified 4 distinct functional trajectories: minimal impairment in 136 participants (41.1%); persistent impairment in 81 (25.4%); partial improvement in 74 (23.5%); and decline in 28 (10.0%). Risk factors for persistent impairment included falls in the 6 months before baseline, depressive symptoms, and low physical performance. Although functional impairment improved in some homeless adults, it persisted or worsened in many others. These findings suggest that, similar to older adults in the general population, functional impairment among older homeless persons is not a transient phenomenon, but instead a chronic issue requiring long-term solutions.

## Introduction

The median age of homeless adults in the U.S. is increasing. Approximately half of single homeless adults are now aged 50 and older, compared to 11% in 1990 [1, 2]. Homeless adults develop aging-related conditions earlier than individuals in the general population, including difficulty with basic activities of daily living (ADLs) that are essential for independence, such as bathing and dressing [3–5]. Difficulty performing these activities, often called “functional impairment,” occurs in 30% of homeless adults in their fifties and early sixties, a prevalence similar to that of housed adults 20 years older [4, 5]. For this reason, homeless adults aged 50 and older are often called “older” despite their relatively young age [3, 4, 6].

Despite this high prevalence of functional impairment, little is known about functional trajectories among homeless adults, including the extent to which these trajectories are similar to those of adults in the general population. Studies of community-dwelling middle-aged and older adults show that function is dynamic: while some functional impairments persist and worsen over time, others, such as those due to injuries, may resolve [7]. In the general population, persistent and worsened functional impairment is strongly associated with higher rates of acute care use [8], institutionalization [9], and death [10, 11]. However, middle-aged people in the general population are more likely to improve after an initial episode of functional impairment than are older adults [12, 13]. It is thought that functional impairment is more transient among middle-aged people because impairments that develop earlier in the life course are more related to acute injuries or illnesses than the more chronic and progressive impairments among older adults [14].

Because homeless people develop functional impairment earlier in the life course [3, 5], it is possible that these impairments would be more transient, similar to middle-aged people in the general population [14]. However, homeless people have a disproportionate prevalence of chronic illnesses and geriatric conditions that are known to be risk factors for functional impairment in the general population [5, 15]. Furthermore, people experiencing homelessness often have great difficulty modifying their environment, and thus may experience a mismatch between their abilities and environment that results in functional impairment or exacerbates an existing impairment [16]. For these reasons, it is possible that functional impairments among homeless older adults often persist and worsen over time, similar to older adults in the population.

Additionally, little is known about risk factors for functional impairment among people experiencing homelessness. Although risk factors for functional impairment among community-dwelling older adults have been well-studied, some hypothesize that homeless persons may have non-traditional risk factors that are prevalent among or unique to homeless people [17]. In her disablement framework, Verbrugge posits that factors both at the level of the individual and at the level of the environment impact an individual’s progression along a central pathway leading to disability [18]. Individual factors may include sociodemographic characteristics, health status, and health-related behaviors, while environmental factors include characteristics of one’s social and physical environment. Among older adults in the general population, risk factors for functional impairment at the level of the individual include female sex, multi-morbidity, depression, cognitive impairment, sensory impairment, smoking, obesity, and lower extremity weakness, while environmental risk factors include social isolation and poor neighborhood safety [15, 19]. However, homeless persons may experience differing risk factors across both the individual and environmental domains, including substance use disorders, limited access to health care, and longer duration of homelessness.[20]

Understanding functional trajectories and risk factors for these trajectories among homeless older adults is key to developing appropriate services and supports to address the needs of this growing population. If most functional impairment among homeless people is transient, there would be less concern about the risk of early nursing home placement. However, if functional impairment persists and worsens over time, services and supports would need to be adapted to address these impairments. Similarly, if most risk factors for functional impairment among homeless persons are like those in the general population, existing interventions could potentially be adapted to address functional impairments. However, differing risk factors may require novel approaches. A key platform for delivering interventions for people experiencing homelessness is permanent supportive housing (PSH), defined as subsidized housing with closely linked or on-site supportive services, which maintains housing among chronically homeless persons [21–23]. Refining the current model of PSH could allow homeless people with chronic functional impairments to be rehoused in community settings, avoiding premature and costly institutionalization.

We identified trajectories of functional impairment and predictors of functional trajectories, including both traditional and non-traditional risk factors, in a prospective cohort study of 350 homeless older adults in Oakland, California. We hypothesized that functional impairment would persist or worsen in most participants. We further hypothesized that among individuals who did experience persistent or worsened functional impairment, non-traditional risk factors including binge drinking, drug use disorders, limited access to health care, and longer duration of homelessness would be important drivers of poorer functional trajectories.

## Materials and methods

### Ethics statement

The institutional review board of the University of California, San Francisco, approved the study (CHR 12–09490). Written consent was obtained from participants.

### Design overview

We conducted a 3-year prospective cohort study of 350 homeless adults aged 50 and older recruited via population-based sampling in Oakland, California (the Health Outcomes in People Experiencing Homelessness in Older Middle agE [HOPE HOME] study). We interviewed participants at baseline and every 6 months thereafter.

### Setting and participants

We sampled homeless individuals using a purposive population-based approach [5, 24]. Sampling locations included low-cost meal programs, overnight shelters, a recycling center, and places where unsheltered people stayed. We set total recruitment targets for each location based on best estimates of the number of unique individuals who visited a site or were unsheltered annually. Individuals meeting a brief eligibility screen were invited to participate in an enrollment interview.

Study staff conducted enrollment interviews from July 2013 through June 2014 at a non-profit community-based organization in Oakland, which provided space for the study. Eligibility criteria included age 50 years or older, English-speaking, currently homeless based on the federal Homeless Emergency Assistance and Rapid Transition to Housing (HEARTH) Act [25], and able to provide informed consent.[26] Participants received $25 gift cards from a major retailer for the enrollment interview and $15 for follow-up interviews.

We conducted follow-up interviews every 6 months for a total of 36 months. To maximize follow-up rates, we conducted monthly check-ins with participants by telephone or in-person. We classified participants as missing for a follow-up if they did not complete an interview within 3 weeks before and 8 weeks after the scheduled follow-up date.

### Measures

#### Outcome: Functional impairment

At baseline and each follow-up, participants reported if they had difficulty performing 5 ADLs (bathing, dressing, eating, transferring, toileting).[27] Individuals who reported having difficulty with an activity were asked if they were unable to complete that activity. We defined an ADL impairment as reporting difficulty with one of these tasks. The primary outcome was the total number of impairments reported at each follow-up (range, 0–5).

#### Traditional risk factors for functional impairment: Individual

To identify characteristics associated with differing functional trajectories, we used covariates assessed at baseline. Sociodemographic characteristics included self-reported age, sex, race/ethnicity, marital/partner status, educational attainment, working for pay in the past 30 days, and median income in the past 30 days from all sources, including wages and benefits.

Measures of health status included self-report of nine physician-diagnosed medical conditions, including hypertension, cardiac disease, congestive heart failure, stroke or transient ischemic attack, diabetes, chronic lung disease, HIV or AIDS, cancer, and arthritis.[28] We categorized the total number of conditions as 0–1 versus ≥2 [29, 30]. We defined visual impairment as corrected visual acuity <20/40 [31], and hearing impairment as self-reported difficulty hearing [32]. Using the Brief Instrumental Functioning Scale, we assessed ability to perform 6 instrumental activities of daily living (IADLs), and defined IADL impairment as difficulty performing 1 or more activities (taking transportation, managing medications, managing money, applying for benefits, setting up a job interview, finding a lawyer) [33]. We defined cognitive impairment as a Modified Mini-Mental State Examination score below the 7^th^ percentile (1.5 standard deviations below a reference cohort mean) or inability to complete the assessment.[34] We defined falls as 1 or more self-reported falls in the 6 months before baseline, and depressive symptoms as a Center for Epidemiologic Studies of Depression Scale score ≥16 (range, 0–60) [35]. We assessed physical performance using the Short Physical Performance Battery (SPPB) and defined reduced performance as a score ≤10 (range, 0–12) [36]. We assessed smoking status using the California Tobacco Survey (current, former, never) [37].

#### Traditional risk factors for functional impairment: Social environment

To assess social support, we asked participants to quantify the number of close confidants, defined as anyone in whom the participant could confide. We categorized these using validated categories (0, 1–5, or ≥6) [38].

#### Non-traditional risk factors for functional impairment: Individual

We assessed health-related behaviors including self-reported binge drinking, defined as drinking ≥6 alcoholic beverages on one occasion monthly or more often [39] and problematic drug use, defined as a WHO Alcohol, Smoking, and Substance Involvement Screening Test score ≥4 for cocaine, amphetamines, or opioids (range, 0–39) [40].

#### Non-traditional risk factors for functional impairment: Environment

Participants reported the total length of time they had experienced homelessness as an adult [41]. We assessed living environment at baseline using a follow-back residential calendar [42]. Participants reported where they had stayed over the past 6 months and the number of days spent in each location. Locations included homeless shelters, unsheltered places, housing belonging to family or friends, transitional housing, hotels or single occupancy units, medical facilities, drug treatment facilities, jail or prison, rooms or apartments that participants rented, and homes that participants owned. We used cluster analysis to categorize these data and identify each participant’s primary living environment [41]. To assess health care access, participants reported if they had a regular location to obtain care other than the emergency department.

### Statistical analyses

We examined participant characteristics at baseline using descriptive statistics. To identify the primary living environment for each participant at baseline, we used cluster analysis, consistent with our previous research [5, 41]. Cluster analysis generates similar groups of participants within a dataset by identifying existing patterns in the data [43, 44]. We used two cluster analysis methods to identify the living environment group as detailed in our previous research [5, 41]. Briefly, this included primary analyses using Ward’s linkage to minimize the sum of squares difference between groups [45], followed by visual analysis of a dendrogram representing the data structure to identify the optimal number of clusters. We verified these cluster classifications using k-medians cluster analysis [46, 47]. We measured the distinctness of the groups generated by these two cluster methods using the pseudo-t^2^ and pseudo-F stopping rules [46].

To identify differing functional trajectories, we used trajectory modeling [48], which estimates multiple probability distributions within longitudinal data [48]. We conducted analyses using the Traj procedure [48], which uses the maximum-likelihood method to fit a semiparametric (discrete) mixture model to longitudinal data. Using a zero-inflated Poisson distribution, we modeled the number of ADL difficulties reported over follow-up. We considered participants who died to have difficulty in all 5 ADLs from the date of death. We performed two sensitivity analyses to assess the stability of the trajectories across differing assumptions. In the first, we excluded participants who died. In the second, we considered both difficulty and inability to perform ADLs, using a summed ADL score (0 [independent], 1 [difficulty performing], or 2 [unable to perform]). The score for all 5 ADLs ranged from 0 to 10.

To identify baseline characteristics that predicted membership in each functional trajectory, we first used bivariable multinomial logistic regression to determine the association of potential risk factors with each trajectory [49], using the minimal impairment group as the reference. We then used multivariable multinomial logistic regression to determine the adjusted association of potential risk factors with each outcome. In the multivariable model, we included characteristics associated with a bivariable multinomial Type 3 p-value < .10. We retained all covariates in the final model. We conducted analyses using Stata 13 (StataCorp, College Station, TX) and SAS 9.4 (SAS Institute, Cary, NC).

## Results

**Fig 1:**
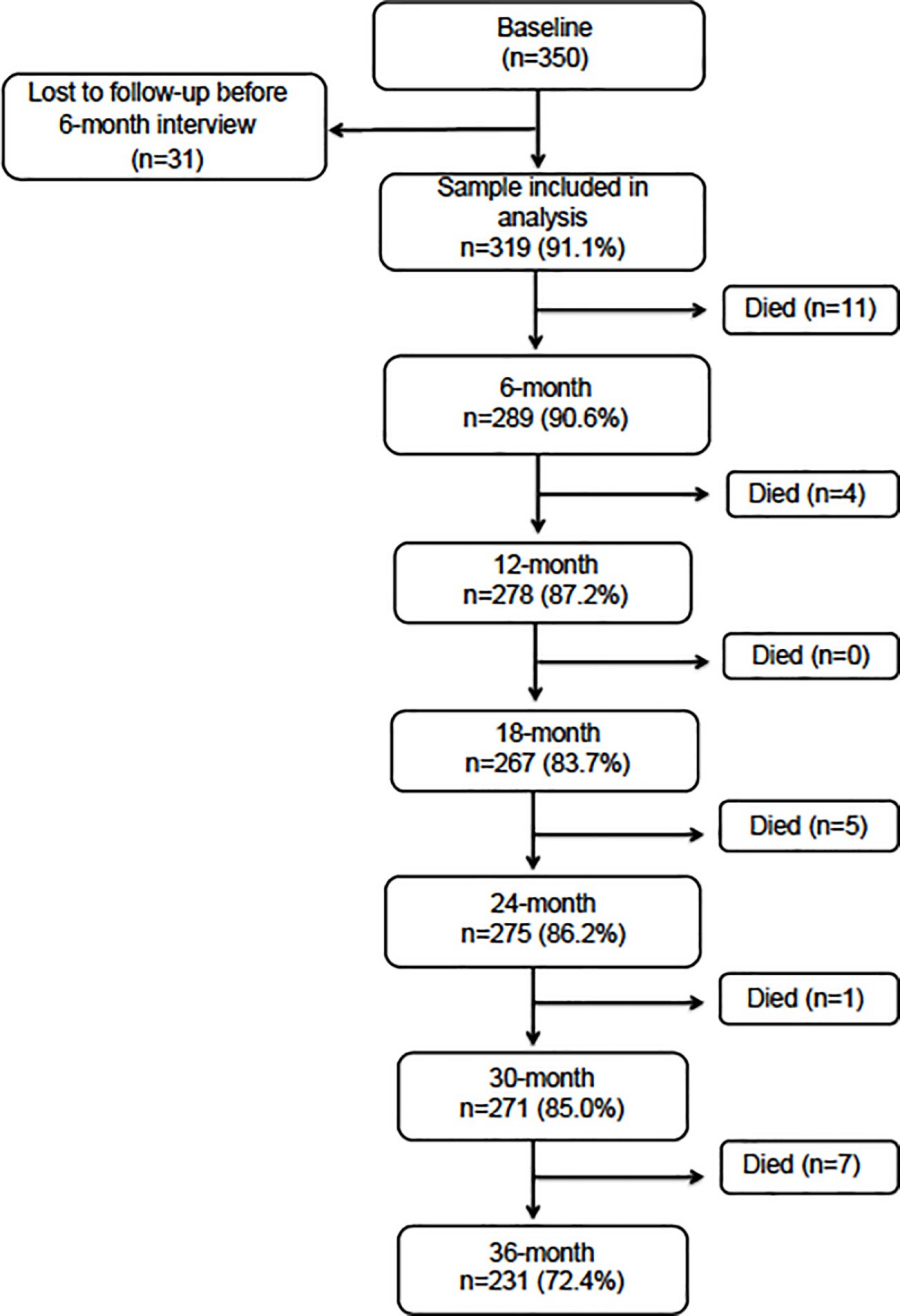
Flow-chart of follow-up of 350 homeless older adults. The figure shows the number of individuals enrolled at baseline and followed at 6-month intervals over 36-months’ follow-up. Deaths between each follow-up are noted.

Of 350 participants who completed a baseline interview, 31 were alive at 6 months but lost to follow-up, leaving 319 participants in whom functional trajectories could be ascertained (Fig 1). Participants lost to follow-up before 6 months were more likely to have depressive symptoms (60.0% vs. 36.3%, p = .01) and to have experienced falls (51.6% vs. 32.0%, p = .03). A higher proportion of men than women were lost to follow-up, but this difference did not reach statistical significance (24.1% vs. 9.7%, p = .07).

### Baseline characteristics of cohort

The mean age of participants was 58.2 years (standard deviation [SD], 5.3), 75.9% were men, 80.9% were African American, and 24.8% had not completed high school nor obtained a GED (Table 1). The median total years of adult homelessness was 2.0 (range, 0.01–42.0 years; interquartile range [IQR], 0.6–8.0). Cluster analysis of the locations where participants stayed during the past 6 months resulted in 4 groupings, as previously reported [41]. The majority of participants spent most nights unsheltered (n = 147). A second group moved between multiple locations including homeless shelters, unsheltered locations, hotels, and jails (n = 79). A third group spent most nights staying with family and/or friends (n = 55), and a fourth group, which had only recently become homeless, spent most nights in rental housing (n = 38). Over half of participants (54.9%) reported 2 or more chronic medical conditions. Functional impairment was common: 12.2% of participants reported difficulty with one ADL, and 26.3% reported difficulty with 2 or more. Geriatric conditions were also common, including visual impairment (46.4%), hearing impairment (36.0%), IADL impairment (48.6%), cognitive impairment (24.8%), falls in the prior 6 months (32.0%), and depressive symptoms (51.4%). More than half of participants (57.8%) had reduced physical performance (SPPB score ≤10).

**Table 1:** Baseline characteristics of 319 homeless participants by functional trajectory.

	All participants (N = 319)	Minimal functional impairment (N = 136)	Persistent functional impairment (N = 81)	Partial functional Improvement (N = 74)	Functional decline (N = 28)	P value^a^
**Traditional individual risk factors**						
**Sociodemographics**						
Age, years mean (SD)	58.2 (5.3)	57.6 (5.2)	58.5 (6.0)	58.6 (5.0)	58.8 (5.6)	0.27
Men, N (%)	242 (75.9)	111 (81.6)	56 (69.1)	57 (77.0)	18 (64.3)	0.09
Black race/ethnicity, N (%)	258 (80.9)	114 (83.8)	63 (77.8)	58 (78.4)	23 (82.1)	0.66
Married or partnered, N (%)	28 (8.8)	11 (8.1)	6 (7.4)	7 (9.5)	4 (14.3)	0.71
Less than high school education, N (%)	79 (24.8)	31 (22.8)	24 (29.6)	16 (21.6)	8 (28.6)	0.59
Worked for pay in past 30 days, N (%)	33 (10.3)	21 (15.4)	4 (4.9)	7 (9.5)	1 (3.6)	0.050
Income in past 30 days, $ median	636 (145, 950)	520 (145, 905)	854 (149, 950)	635 (145, 950)	431 (210, 879)	0.33
**Health status, N (%)**						
2 or more chronic medical conditions	175 (54.8)	53 (39.0)	57 (70.4)	49 (66.2)	16 (57.1)	<0.001*
Visual impairment	143 (46.4)	56 (43.1)	35 (44.9)	37 (51.4)	15 (53.6)	0.58
Hearing impairment	114 (36.0)	36 (26.7)	36 (44.4)	35 (47.9)	7 (25.0)	0.003*
ADL impairment						<0.001*
1 impairment	39 (12.2)	5 (3.7)	11 (13.6)	17 (23.0)	6 (21.4)	
≥2 impairments	84 (26.3)	0	56 (69.1)	28 (37.8)	0	
IADL impairment	155 (48.6)	57 (41.9)	51 (63.0)	37 (50.0)	10 (35.7)	0.01*
Cognitive impairment	79 (24.8)	33 (24.3)	17 (21.3)	20 (27.0)	9 (32.1)	0.67
Falls in past 6 months	10 (32.0)	28 (20.6)	43 (53.1)	21 (28.4)	10 (35.7)	<0.001*
Depressive symptoms	163 (51.4)	57 (41.9)	53 (66.3)	38 (52.1)	15 (53.6)	0.007*
Body mass index						
<18	8 (2.6)	2 (1.5)	3 (3.9)	1 (1.4)	2 (7.7)	0.24
18–24.99	117 (38.4)	49 (37.4)	26 (34.2)	32 (44.4)	10 (38.5)	
25–29.99	96 (31.5)	48 (36.6)	20 (26.3)	23 (31.9)	5 (19.2)	
≥30	84 (27.5)	32 (24.4)	27 (35.5)	16 (22.2)	9 (34.6)	
SPPB score ≤10	181 (57.8)	55 (40.4)	63 (81.8)	44 (61.1)	19 (67.9)	< .001
**Smoking status, N (%)**						
Current smoker	204 (63.9)	79 (58.1)	54 (66.7)	52 (70.3)	19 (67.9)	0.36
Former smoker	40 (12.5)	21 (15.4)	9 (11.1)	5 (6.8)	5 (17.9)	
**Traditional environmental risk factors**						
**Social Support, n(%)**						
No confidants	97 (30.5)	42 (31.1)	27 (33.3)	22 (29.7)	6 (21.4)	0.71
1–5 confidants	191 (60.1)	78 (57.8)	47 (58.0)	45 (60.8)	21 (75.0)	
6+ confidants	30 (9.4)	15 (11.1)	7 (8.6)	7 (9.5)	1 (3.6)	
**Novel individual risk factors**						
Binge drinking	72 (22.6)	34 (25.0)	15 (18.5)	12 (16.4)	11 (39.3)	0.06
Drug use problem	158 (49.5)	61 (44.9)	47 (58.0)	36 (48.6)	14 (50.0)	0.31
**Novel environmental risk factors**						
Years homeless as adult, median (IQR)	2.0 (0.6, 8.0)	1.6 (0.5, 7.5)	2.0 (0.7, 9.5)	2.0 (0.6, 6.0)	5.5 (1.3, 9.4)	0.16
**Living environment, N (%)**						
Unsheltered	147 (46.1)	61 (44.9)	42 (51.9)	36 (48.7)	8 (28.6)	0.18
Users of multiple locations	79 (24.8)	30 (22.1)	18 (22.2)	22 (29.7)	9 (32.1)	
Cohabiters	55 (17.2)	22 (16.2)	13 (16.1)	12 (16.2)	8 (28.6)	
Recently homeless	38 (11.9)	23 (16.9)	8 (9.9)	4 (5.4)	3 (10.7)	
**Health services access**						
Regular healthcare location, N (%)	234 (73.3)	95 (69.9)	65 (80.2)	54 (73.0)	20 (71.4)	0.41

Abbreviations: ADL, activities of daily living; IADL, instrumental activities of daily living; SPPB, Short Physical Performance Battery

^a^All p-values represent chi-square tests for homogeneity among the four trajectory groups, with the exception of the p-values for age, median income in the past 30 days, and total years homelessness, which represent the Kruskal-Wallis test

*P values marked with an asterisk are statistically significant at p < .05.

One-fifth of participants (22.6%) reported binge drinking, 49.5% reported problematic drug use, and 63.9% were current smokers. Nearly three quarters reported a regular location for health care (73.3%).

### Trajectories of functional impairment

**Fig 2:**
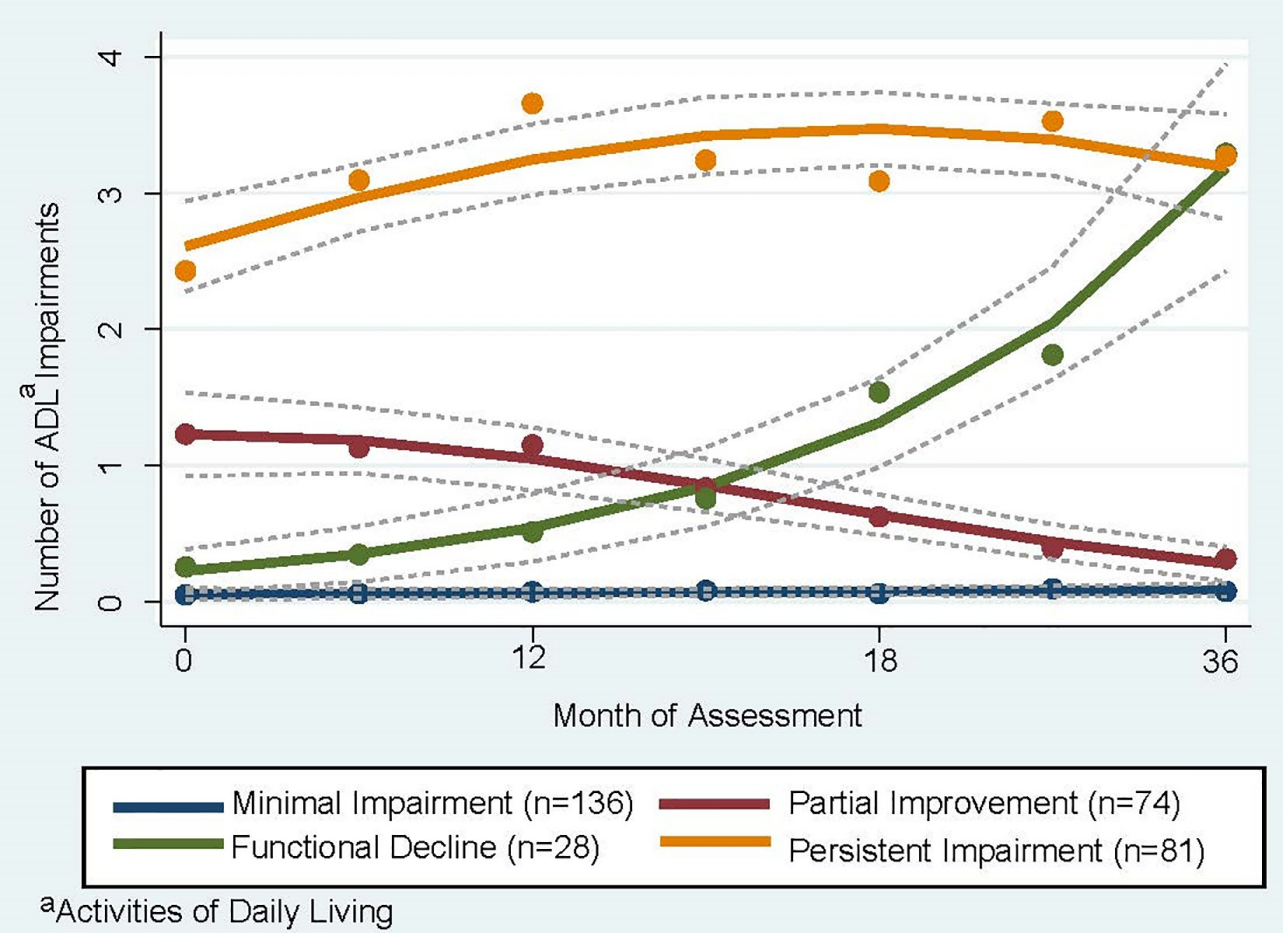
Functional trajectories among homeless older persons over the course of three-year follow-up. We used trajectory modeling to identify three-year functional trajectories based on participants’ self-reported ability to perform 5 basic activities of daily living (ADLs). We considered participants who died over follow-up to have difficulty in all 5 activities of daily living from the date of death. Number of ADL difficulties are portrayed on the y-axis, with month of assessment on the x-axis. Dotted lines indicate 95% confidence intervals for each trajectory.

Trajectory modeling yielded 4 distinct trajectories (Fig 2). In the first, participants had minimal functional impairment, with an average number of ADL impairments at baseline of 0.04 (SD, 0.19) compared to 0.07 (SD, 0.33) at follow-up (“minimal functional impairment,” n = 136). In the second, participants had persistent impairment, with an average number of ADL impairments of 2.4 at baseline (SD, 1.6) compared to 3.3 (SD, 1.7) at follow-up (“persistent functional impairment,” n = 81). In the third, participants experienced partial functional improvement, with the average number of ADL impairments decreasing from 1.3 at baseline (SD, 1.3) to 0.3 at follow-up (SD, 0.6); (“partial functional improvement,” n = 74). In the final trajectory, participants experienced functional decline, with the average number of ADL impairments increasing from 0.2 at baseline (SD, 0.4) to 3.4 at follow-up (SD, 1.4); (“functional decline,” n = 28). The results of the sensitivity analyses were similar to the main analysis (see S1 and S2 Figs). Over the 36-month study period, 28 participants died, of whom 8 were in the functional decline group and 20 in the persistent impairment group.

### Baseline predictors of functional trajectories

In bivariable analyses, several traditional, individual-level baseline characteristics were significantly associated with subsequent functional trajectory compared to a reference of minimal impairment (Table 2). Participants in the persistent impairment group were more likely to be women (odds ratio [OR] 1.98; 95% CI, 1.04–3.79), to have two or more chronic conditions (OR 3.72; 95% CI 2.05–6.74), hearing impairment (OR 2.20; 95% CI 1.22–3.96), falls in the prior 6 months (OR 4.36; 95% CI, 2.37–8.02), depressive symptoms (OR 2.72; 95% CI, 1.52–4.86), and reduced physical performance (OR 6.63; 95% CI 3.36–13.08). Participants in the partial functional improvement group were more likely to be women (OR 2.47; 95% CI 1.01–6.04), to have two or more chronic conditions (OR 3.07; 95% CI 1.69–5.58), and to have reduced physical performance (OR 3.11; 95% CI 1.30–7.44). Participants in the functional decline group were more likely to have hearing impairment (OR 2.53; 95% CI 1.39–4.63) and reduced physical performance (OR 2.31; 95% CI 1.28–4.18). Of the non-traditional risk factors, only longer total years of homelessness was more common among individuals in the functional decline group (OR 1.05; 95% CI, 1.01–1.10).

**Table 2:** Association of baseline characteristics with functional trajectories.

	Persistent Functional Impairment, Odds Ratio (95% CI)^a^		Partial Functional Improvement, Odds Ratio (95% CI)^a^		Functional Decline, Odds Ratio (95% CI)^a^	
	Unadjusted	Adjusted	Unadjusted	Adjusted	Unadjusted	Adjusted
**Traditional individual risk factors**						
**Sociodemographics**						
Age	1.03 (0.98–1.09)	1.03 (0.97–1.11)	1.04 (0.97–1.13)	1.03 (0.97–1.10)	1.04 (0.98–1.09)	1.05 (0.96–1.14)
Women	**1.98** **(1.04–3.79)**	2.00 (0.95–4.20)	**2.47** **(1.01–6.04)**	1.21 (0.57–2.57)	1.32 (0.66–2.67)	2.50 (0.98–6.35)
**Health status**						
≥2 chronic conditions	**3.72** **(2.05–6.74)**	1.87 (0.94–3.72)	**3.07** **(1.69–5.58)**	**2.13** **(1.11–4.09)**	2.09 (0.91–4.80)	1.82 (0.73–4.51)
Hearing impairment	**2.20** **(1.22–3.96)**	1.60 (0.81–3.20)	0.92 (0.36–2.36)	**2.12** **(1.10–4.06)**	**2.53** **(1.39–4.63)**	0.68 (0.25–1.85)
Falls in past 6 months	**4.36** **(2.37–8.02)**	**2.84** **(1.43–5.61)**	1.53 (0.79–2.96)	1.16 (0.56–2.40)	2.14 (0.88–5.20)	1.59 (0.62–4.05)
Depressive symptoms	**2.72** **(1.52–4.86)**	**3.02** **(1.49–6.16)**	1.60 (0.70–3.65)	1.60 (0.82–3.11)	1.50 (0.84–2.68)	1.39 (0.56–3.48)
Short Physical Performance Battery score ≤10	**6.63** **(3.36–13.08)**	**4.77** **(2.30–9.91)**	**3.11** **(1.30–7.44)**	**2.02** **(1.06–3.86)**	**2.31** **(1.28–4.18)**	2.39 (0.96–5.97)
**Novel individual risk factors**						
Binge drinking	0.68 (0.34–1.45)	0.56 (0.25–1.24)	1.94 (0.82–4.59)	0.49 (0.21–1.10)	0.59 (0.28–1.23)	1.94 (0.77–4.90)
**Novel environmental risk factors**						
Years homeless as adult	1.02 (0.98–1.10)	1.00 (0.96–1.04)	0.99 (0.94–1.03)	0.98 (0.93–1.02)	**1.05** **(1.01–1.10)**	1.04 (0.99–1.10)

^a^The referent group is the group with minimal impairment in activities of daily living.

Bolded results indicate odds ratios that are statistically significant.

After multivariable adjustment, traditional individual-level characteristics predicted subsequent functional trajectory. Participants in the persistent impairment group had a higher adjusted odds of falls in the prior six months (adjusted OR [AOR] 2.84; 95% CI, 1.43–5.61), depressive symptoms (AOR 3.02; 95% CI, 1.49–6.16), and reduced physical performance (AOR, 4.77; 95% CI, 2.30–9.91) (Table 2). Participants in the partial functional improvement group had a higher adjusted odds of having two or more chronic conditions (AOR 2.13; 95% CI, 1.11–4.09), hearing impairment (AOR 2.12; 95% CI, 1.10–4.06), and reduced physical performance (AOR 2.02; 95% CI, 1.06–3.86). After multivariable adjustment, bivariable predictors of functional decline were no longer statistically significant, though being a women (AOR 2.50; 95% CI, 0.98–6.35) and having reduced physical performance (AOR 2.39; 95% CI, 0.96–5.97) were associated with functional decline at borderline significance. None of the non-traditional characteristics were significantly associated with functional trajectory.

## Discussion

In this population-based cohort study of homeless adults aged 50 and over, we found that functional trajectories varied substantially. While a majority of participants had either minimal functional impairment or partial functional improvement over three-year follow-up, more than a third experienced persistent or worsened impairment. Several baseline characteristics were significantly associated with persistent impairment, including depressive symptoms, low physical performance, and falls. Our findings suggest that a substantial proportion of homeless older adults experience non-transient functional impairment, and that housing and healthcare interventions for this population must include accommodations to address these impairments.

Previous cross-sectional studies show that homeless adults develop functional impairment earlier than individuals in the general population [3–5], but little is known about the trajectories of these impairments. We hypothesized that these trajectories would be relatively similar to those among older adults for two main reasons. First, homeless older adults have a disproportionate prevalence of chronic conditions and geriatric conditions that are known risk factors for functional impairment in the general older population [5, 15], and second, homeless people may have great difficulty modifying their environment to match their abilities [5]. Consistent with this hypothesis, we found that more than one third of participants experienced persistent or worsened impairment. These findings are consistent with research showing that functional impairment among community-dwelling older adults is dynamic: while nearly 80% of impairments resolve, 20% persist or worsen over time [50–52]. Recovery from functional impairment is often short-lived, however; individuals who initially recover from disability are at high risk for chronic disability [51]. The same may be true of homeless participants who experienced partial functional improvement, although longer follow-up is needed to test this hypothesis.

We identified several risk factors for persistent functional impairment, many of which are similar to established risk factors in community-dwelling older adults. Depressive symptoms strongly predicted persistent functional impairment. Depression is a well-established risk factor for disability in the general population, and is thought to impact function by eroding the physical skills needed to maintain independence and increasing vulnerability to stressors that can accelerate functional decline [53, 54]. Depression is far more prevalent among homeless people than community-dwelling adults [4], and may represent an important target for strategies to improve function in this population. Of note, because participants had functional impairment at baseline, it is possible that impairment preceded depression in some participants [55].

Falls were an important risk factor for persistent functional impairment. In the general population, falling increases the risk for functional impairment through complex pathways including injury, loss of confidence performing daily tasks, subsequent deconditioning, and worsening balance and gait [56]. Falls are highly prevalent among homeless persons, likely due to a high burden of risk factors including medical comorbidities, substance use problems, and environmental exposures [17]. Addressing risk factors for falls, including housing interventions that address the mismatch between one’s abilities and environment, may play a key role in improving function among homeless persons.

Reduced physical performance predicted both persistent functional impairment and functional improvement. These findings are consistent with research in the general population showing that physical performance strongly predicts function [36] and may lie on the causal pathway to functional impairment [18, 36]. Physical performance can be improved through physical activity [57], providing another potential target for improving function among homeless persons.

Lastly, having multiple chronic conditions predicted partial functional improvement. In contrast, multi-morbidity increases the risk for functional decline among community-dwelling older adults [15]. While several factors may explain this difference, homeless people experience a disproportionate burden of symptoms that are related to chronic illness and may impact function, such as joint pain from arthritis [58]. Our findings may reflect transient improvements in such symptoms.

Contrary to our hypothesis, non-traditional risk factors for functional impairment–binge drinking, drug use problems, duration of homelessness, and access to health care–did not significantly predict functional trajectory. While longer duration of homelessness and binge drinking were both associated with an increased odds of functional decline, the results were not statistically significant. Although these findings suggest that exposure to homelessness and substance use problems may play a role in precipitating functional decline among homeless persons, a larger sample size may be needed to further evaluate these risk factors.

These findings have implications for delivery of services and supports to homeless older adults. In the general population, approaches to managing functional impairment are multi-factorial, including environmental modification, physical rehabilitation, and addressing risk factors such as depression and falls. The trajectories and risk factors for functional impairment in our cohort are similar to those of community-dwelling older adults, suggesting that similar approaches may be effective among homeless people. However, these approaches cannot be effectively implemented in the environments where homeless people live.

Currently, supplies of Permanent Supportive Housing (PSH) are limited and not adapted to the needs of an aging population, and thus many homeless and formerly homeless people with functional impairment may be prematurely institutionalized [59]. While the optimal approach to adapting PSH for an aging population is not yet known, preliminary evidence suggests that PSH that incorporates environmental modifications and personal care attendants may be a cost effective and less restrictive alternative to placing homeless people with functional impairment in nursing homes [60, 61]. Among low-income Medicare beneficiaries in the general population, a home-based intervention including home repair and occupational therapy improved function and delayed nursing home admission [62]. These findings suggest that appropriately adapted PSH, incorporating environmental modifications, rehabilitation, and accessible personal care, could allow individuals with functional impairment to age in place while delaying or preventing the need for nursing home care.

This study has several limitations. We excluded individuals in whom we could not ascertain functional trajectories, including participants lost to follow-up before 6 months. Individuals lost to follow-up were more likely to have depressive symptoms and falls, and thus our findings may underestimate persistent functional impairment. It is possible that participants assigned to the functional decline group experienced transient decline that would later improve, though our relatively long follow-up period mitigates this concern. Because the sample size for individuals in the functional decline group was relatively small (n = 28), we may have lacked power to detect predictors of this trajectory. We measured function by self-report rather than objective measures. However, self-reported function is a key person-centered measure that strongly predicts poor outcomes [9, 10]. Moreover, we included objective measures of lower extremity function, which strongly predicted self-reported function. Although we assessed living environment at baseline, we did not perform time-varying analyses of housing status over the follow-up period. Because the current study focused on assessing functional trajectories and baseline predictors of those trajectories, a time-varying analysis was beyond the scope of the current study. However, we will explore this issue in future work. The study includes a higher proportion of African Americans than previous cohorts of homeless individuals [63]. However, in the general population, the risk of functional decline is similar across racial/ethnic groups [15], and participant characteristics were otherwise similar to a nationally representative sample [63]. Furthermore, our study employs a population-based design in which we sampled individuals purposively to reflect all individuals experiencing homelessness who are aged 50 or older. Thus, the sample is likely to be more reflective of the source population of homeless older adults than studies focusing on specific populations, such as people who are chronically homeless or using acute care.

## Conclusions

A substantial proportion of homeless older adults experienced persistent or worsened functional impairment. As the population of homeless older adults grows, housing and healthcare interventions must include accommodations to address functional impairments and reduce associated risks of premature institutionalization. Housing programs that incorporate environmental modifications and personal care hold promise for addressing these risks and improving outcomes for this vulnerable and growing population.

## Supporting information

S1 Fig Functional trajectories among homeless older persons, excluding participants who died. We used trajectory modeling to identify three-year functional trajectories based on participants’ self-reported ability to perform 5 basic activities of daily living (ADLs), excluding participants who died. Number of ADL difficulties are portrayed on the y-axis, with month of assessment on the x-axis. Dotted lines indicate 95% confidence intervals for each trajectory.
(EPS)

S2 Fig Functional trajectories among homeless older persons, accounting for difficulty and inability to perform activities of daily living. We used trajectory modeling to identify three-year functional trajectories based on participants’ self-reported ability to perform 5 basic activities of daily living (ADLs), accounting for difficulty and inability to perform ADLs. For each ADL, the score could take a value of 0 (independent), 1 (difficulty performing), or 2 (unable to perform). Number of ADL difficulties are portrayed on the y-axis, with month of assessment on the x-axis. Dotted lines indicate 95% confidence intervals for each trajectory.
(EPS)

S1 Dataset Dataset for baseline characteristics and multinomial models. This file includes the data for the variables in Table 1 and in the multinomial models.
(DTA)

S2 Dataset Dataset for trajectory analyses. This file includes the activities of daily living (ADLs) in wide format for use in the Traj procedure analyses.
(DTA)
